# Supplementary material for: 3D-printed low-voltage-driven ciliary hydrogel microactuators
Source: Nature. 2026 Jan 14;649(8098):885–93. doi: 10.1038/s41586-025-09944-6 (PMC12823445; doi:10.1038/s41586-025-09944-6)
Supplement: Supplementary file 1 — This file contains Supplementary Notes, Supplementary Figures, legends for the Supplementary Videos and Supplementary References. [file 41586_2025_9944_MOESM1_ESM.pdf]

nature portfolio

https://doi.org/10.1038/s41586-025-09944-6

# Supplementary information

# 3D-printed low-voltage-driven ciliary hydrogel microactuators

In the format provided by the authors and unedited

# Supplementary Information for

## 3D-printed low-voltage-driven ciliary hydrogel microactuators

Zemin Liu, Che Wang, Ziyu Ren, Chunxiang Wang, Wenkang Wang, Jongkuk Ko, Shanyuan Song, Chong Hong, Xi Chen, Hongguang Wang, Wenqi Hu and Metin Sitti

Corresponding authors: wenqi@ust.hk; msitti@ku.edu.tr

**The PDF file includes:**

Supplementary Notes
- Note 1: Multi-layer microelectrodes fabrication
- Note 2: Hydrogel solution for molding
- Note 3: Gel microcilia molding
- Note 4: Hydrogel microactuator printing parameter discussion
- Note 5: SEM imaging
- Note 6: TEM imaging
- Note 7: Experiment setup
- Note 8: AFM test
- Note 9: 100-channel control board
- Note 10: Simulation parameters
- Note 11: Analysis between the 2 µm- and 10 µm-diameter hydrogel cilia
- Note 12: Microelectrodes working in high-concentration ionic solutions

Supplementary Figs. 1-11

**Other Supplementary Materials for this manuscript include the following:**

Supplementary Videos 1-31

## Supplementary Notes

### Note 1: Multi-layer microelectrodes fabrication

In comparison to single-layer microelectrodes, fabricating multi-layer microelectrodes requires multiple exposures and alignments on the same sample. These additional steps are designed to prevent short-circuiting and increase electrode density.

Supplementary Fig. 2 shows the fabrication process of multi-layer microelectrodes, which builds upon the completed single-layer microelectrode sample. Below are the step-by-step details:

Step 1. Preparation of the photo-curable polyimide substrate (Supplementary Fig. 2a).

The photo-curable polyimide HD 4100 (HD MicroSystems) is applied between the Pt microelectrode layers to function as insulation blocks. HD 4100 is poured onto a completed single-layer microelectrode substrate, then spin-coated at 3000 rpm for 30 seconds.

Step 2. Soft bake (Supplementary Fig. 2b).

Place the HD 4100 sample on an 85 °C hot plate for 90 seconds, then ramp up the temperature to 95 °C, maintaining it for 90 seconds. The ramp-up rate is 10 °C per minute.

Step 3. UV exposure (Supplementary Fig. 2c).

Align the position of the photomask and HD 4100 substrate to ensure that the exposed region covers the first Pt microelectrode layer. Then expose the HD 4100 substrate for 30 seconds using the MJB4 mask aligner.

Step 4. Post-exposure bake (Supplementary Fig. 2d).

Bake the exposed HD 4100 substrate at 80 °C for 60 seconds.

Step 5. Development (Supplementary Fig. 2e).

Develop the HD 4100 substrate in cyclopentanone (Sigma-Aldrich) for 3 minutes, then rinse the sample in 1-methoxy-2-propanol acetate (PGMEA, Sigma-Aldrich) for 3 minutes. Afterward, rinse the sample in IPA (Sigma-Aldrich) for 3 minutes.

Step 6. Curing the patterned HD 4100 block (Supplementary Fig. 2f).

Place the developed sample on a hot plate and heat it from room temperature to 150 °C. Maintain this temperature for 10 minutes, then increase it to 200 °C. The temperature ramp rate is 20 °C per minute. Hold this temperature for 5 hours to fully cure the patterned HD 4100 polyimide.

Step 7. Fabrication of the second Pt microelectrode layer (Supplementary Fig. 2g).

Repeat the single-layer microelectrode fabrication process on the HD 4100 sample to form the second microelectrode layer. Carefully align the photomask and sample during the UV exposure step.

Supplementary Fig. 4 shows the multi-layer microelectrode substrate. Furthermore, this substrate is employed to control the one-million hydrogel array.

All completed microelectrode substrates, including both single-layer and multi-layer designs, will be placed on a hot plate and heated at 200 °C for 1 hour. The heating process begins at room temperature and ramps up to 200 °C at a rate of 20 °C per minute. Once the target temperature is reached, it is maintained for 1 hour before the hot plate is turned off. The substrates are then allowed to cool on the hot plate. This step is designed to improve the adhesion between the Pt microelectrodes and the underlying PI 2611 layer.

## Note 2: Hydrogel solution for molding

Since ethylene glycol and high concentrations of AAc can soften or dissolve the photoresist mold used in hydrogel molding, the hydrogel solutions for direct printing are unsuitable for molding applications. We switch to a water-based solution to address this limitation and optimize the AAc and AAm mass fractions. The optimization goal is to ensure that the photoresist mold remains intact in the low-pH environment caused by AAc. At the same time, the retrieved hydrogel actuator can still respond to an electric signal. After several optimization trials, we identify the following hydrogel solution for molding. The mass fractions are based on the initial mass of DI water.

Hydrogel solution for molding: AAc 6 wt%, AAm 1.5 wt%, BIS 0.4 wt%, Lithium phenyl-2,4,6-trimethylbenzoylphosphinate (photoinitiator, Sigma-Aldrich) 0.1 wt%.

## Note 3: Gel microcilia molding

Supplementary Fig. 3 depicts the fabrication flow of the one-million molded hydrogel array. The following are the details of the molding method:

Step 1. Photoresist coating (Supplementary Fig. 3a).

Pour thick positive photoresist AZ IPS 6090 onto a multi-layer microelectrode substrate, then spin-coat the substrate at 2500 rpm for 10 seconds.

Step 2. Soft bake (Supplementary Fig. 3b).

Place the photoresist-coated substrate on an 80 °C hot plate for 3 minutes. Gradually ramp up the temperature to 125 °C and hold it for 3 minutes.

Step 3. UV exposure (Supplementary Fig. 3c).

Align the photomask approximately with the electrode substrate to ensure that the photomask pattern region is centered on the substrate. Expose the substrate for 36 seconds using the MJB4 mask aligner.

Step 4. Post-exposure bake (Supplementary Fig. 3d).

Bake the exposed substrate at 100 °C for 100 seconds.

Step 5. Development (Supplementary Fig. 3e).

Develop the AZ IPS 6090-coated substrate in AZ 726 developer for 150 seconds to form the hydrogel pillar cavity. The developed substrate is used as the mold for hydrogel molding.

Step 6. Filling the mold with hydrogel solution (Supplementary Fig. 3f).

Pour the water-based hydrogel solution onto the mold surface, then place it in a vacuum desiccator for 5 minutes to fill the cavities.

Step 7. UV flood-exposure to cure the hydrogel structure (Supplementary Fig. 3g).

Place the hydrogel-filled substrate in a 365 nm UV box for 30 minutes to cure the hydrogel structure.

Step 8. Retrieving the hydrogel pillar array from the mold (Supplementary Fig. 3h).

Immerse the substrate in a stripper bath at 55 °C for 30 minutes to dissolve the photoresist mold. Then transfer the sample to a DI water bath. The stripper is a mixture of ethylene glycol (10 wt%) and ethanol (90 wt%). Note that the commercial stripper TechniStrip Micro D350 is too strong and may damage the soft, sensitive hydrogel structure.

Supplementary Fig. 5 provides a top view of the developed mold and a cross-sectional view of the mold, and the retrieved hydrogel cilia are shown in Supplementary Fig. 6.

**Note 4: Hydrogel microactuator printing parameter discussion**

The piezo scan mode is employed for the hydrogel printing in this work. The slicing and hatching distances are set to 300 nm and 200 nm, respectively, with a 45° hatching angle between adjacent layers (Extended Data Fig. 3). These parameters are optimized to produce microscale hydrogel actuators with network pores of approximately 20–80 nm (Extended Data Fig. 1b(ii)).

The formation of sub-100-nm pores could arise from the synergistic interplay between the slicing and hatching parameters and the intrinsic three-stage mechanism of 2PP. During the initiation phase, localized laser irradiation decomposes the photoinitiator, generating free radicals. In the growth phase, these radicals react with monomers to form polymeric chains, extending the polymerized region beyond the laser's focal voxel through radical diffusion and chain propagation. In the termination phase, radical recombination completes the crosslinked hydrogel network[1]. Together, the controlled slicing and hatching parameters and the growth dynamics of 2PP cooperatively define the nanoscale pore architecture within the printed hydrogel microactuators.

The printing system integrates a high-precision piezoelectric stage (P-563.3CD, Physik Instrumente (PI) SE & Co. KG) that controls the slicing and hatching steps with a resolution of 0.5–2 nm[2], ensuring accurate positioning and layer alignment. This level of precision indicates that the current system is fully capable of further optimizing the hydrogel network architecture if required. The printing parameters reported here may require slight adjustment when using other systems.

**Note 5: SEM imaging**

The completed hydrogel is transferred from DI water to ethanol. To preserve the hydrogel geometry during this process, ethanol baths with increasing concentrations (20 wt%, 40 wt%, 60 wt%, 80 wt%, and 100 wt%) are prepared. In each bath, the sample is immersed for 30 minutes before being transferred to the next higher ethanol concentration. Following the ethanol exchange process, the sample is dried using a critical point dryer (Leica EM CPD 300). Subsequently, a thin layer of Au is deposited on the sample via sputtering, and imaging is performed using scanning electron microscopy (SEM; Zeiss, Gemini).

**Note 6: TEM imaging**

Arrays of thin film hydrogels are printed and transferred to the Cu TEM sample holder. Then the whole sample is dried using the same protocol mentioned above. The imaging is performed using transmission electron microscope (TEM; ARM200F)

**Note 7: Experiment setup**

An upright microscope (Zeiss) and a high-speed camera (Phantom) are used to record experimental videos. A computer-controlled board (Supplementary Fig. 8) to generate the actuation signals for the hydrogel cilia.

Hydrogel cilia actuators used for step response (Fig. 2a-b) characterization in both DI water and NaCl solutions have identical geometries, with a diameter of 10 µm and a height of 90 µm. The applied electric field intensity is 10,000 V/m. Four kinds of hydrogel cilia (AAc 15 wt%, AAc 30 wt%, AAc 45 wt%, AAc 60 wt%) are tested in DI water. And the hydrogel cilia with 30 wt% AAc are used for step response in four NaCl solutions of different concentrations (0.00769 mol/L, 0.0154 mol/L, 0.0192 mol/L, 0.1538 mol/L). The applied electric field intensity during these tests is 10,000 V/m. The typical step response in 0.00769 mol/L and 0.1538 mol/L NaCl solutions are shown in Fig. 2

For the dynamic response test, a controllable square-wave signal generated by the control board is used for actuation. The square wave has a peak voltage of 1.5 V and a valley voltage of 0 V, with a 50% duty cycle. The electric field intensity is controlled by the distance between the microelectrodes (microelectrodes with different distances are fabricated for characterization usage), while the peak square-wave voltage is kept constant at 1.5 V to avoid electrochemical reactions in the ionic aqueous environment. For the AAc concentration (AAc 15 wt%, AAc 30 wt%, AAc 45 wt%, AAc 60 wt%) dynamic response tests (Fig. 2c(i)), all hydrogel cilia actuators have the same geometry (10 µm diameter, 90 µm height), the applied electric field intensity is 10,000 V/m, and the aqueous environment is DI water. For the durability test (Fig. 2c(ii)), the same 30 wt% AAc hydrogel cilium actuator is used. This cilium is continuously actuated at 20 Hz for approximately five hours in the DI water, with an electric field intensity of 22,000 V/m. For the diameter test (Fig. 2c(iii)), hydrogel cilia with diameters of 2 µm (18 µm height) and 10 µm (90 µm height) are fabricated using the same 30 wt% AAc hydrogel solution. Both are tested under the same electric field intensity (20,000 V/m) and working solution (DI water). For the solution

(DI water, physiological saline, DPBS, human saliva, human serum, and mouse plasma) test (Fig. 2c(iv)), 2 µm diameter hydrogel cilia with an 18 µm height (30 wt% AAc) are tested under an electric field intensity of 20,000 V/m.

For the reprogrammable motion experiments, four hydrogel cilia systems are used. A 2 gel microcilia array (cilium diameter: 2 µm, height: 18 µm, AAc 30 wt%) works in physiological saline (Fig. 3a). A 5×5 gel microcilia array (cilium diameter: 10 µm, height: 90 µm, AAc 30 wt%) works in DI water (Fig. 3b). A 625 gel microcilia array (cilium diameter: 10 µm, height: 90 µm, AAc 30 wt%) works in DI water (Fig. 3c). The artificial star larvae works in 0.00769 mol/L NaCl. The flapping micromachine works in DI water (Fig. 3d).

The solution used in fluid experiments is a low-ion-concentration solution (0.00769 mol/L NaCl). The hydrogel cilia used here have a diameter of 10 µm, a height of 90 µm, and 30 wt% of AAc. For flow visualization, 1 µm diameter polystyrene particles (PS-FluoRed-1.0, Micro Particles GmbH) are added to the solution.

**Note 8: AFM test**

To investigate the influence of hydrogel monomer concentration on modulus, we print four types of hydrogel solutions: AAc 15 wt%, AAc 30 wt%, AAc 45 wt%, and AAc 60 wt%. The modulus is tested using an atomic force microscope (AFM, JPK Instruments) equipped with a round tip (CP-FM-BSG-A-5, Nano and More GmbH).

**Note 9: 100-channel control board**

The half-bridge 10×10 array circuit outputs 100 channels of digital voltage, each channel individually controllable. The output voltage range is adjustable between 1 V and 5 V, depending on the input voltage. The circuit comprises a microcontroller, GPIO expanders, and a half-bridge driver array. The Arduino Uno R3 microcontroller communicates with the GPIO expander chips via an $I^2C$ bus. Each MCP23017 GPIO expander chip is assigned a unique $I^2C$ device address. A total of six MCP23017 chips and four Arduino pins are used to control the 100 half-bridge circuits. The digital input signals govern each half-bridge driver, enabling either 0 V or the input voltage outputs.

**Note 10: Simulation parameters**

The hydrogel mechanism bending governing equations provided in the main text Methods section are numerically solved by the commercial software COMSOL in a coupled way. Following are the simulation details.

To reduce simulation time, the hydrogel cilia actuation system is simplified to a 2D model, as shown in Supplementary Fig. 9a. The dimensions of this 2D model are 200 µm (width) by 150 µm (height). The hydrogel region is located between 95 µm and 105 µm, with a height of 90 µm. The hydrogel region is shaded in blue, while the light red shaded area represents the aqueous environment. In experiments, the hydrogel cilia working bath measured 4 cm (length) by 4 cm (width) by 3 mm (height). Extended Data Fig. 6b shows a cross-sectional view of the working bath, with a zoomed-in view highlighting the hydrogel cilia working environment. During actuation, ions in the working bath could freely move in the small actuation region, which follows the governing equations. All mobile ions are assumed to have the same diffusion coefficient and ion mobility parameters in the hydrogel region and the surrounding aqueous environment for that hydrogel is a high water content material.

The electric potential is applied at nodes (0, 0) and (200, 0), as shown in Supplementary Fig. 9a. Supplementary Fig. 9b illustrates the potential distribution in the initial state. The diffusion coefficients of the mobile ions used in the simulation are as follows: hydrogen ion $D_{H^+} = 9.31 \times 10^{-9} m^2 s^{-1}$, sodium ion $D_{Na^+} = 1.33 \times 10^{-9} m^2 s^{-1}$, hydroxide ion $D_{OH^-} = 5.27 \times 10^{-9} m^2 s^{-1}$, and chloride ion $D_{Cl^-} = 2.03 \times 10^{-9} m^2 s^{-1}$. The ions' mobility data are: $\mu_H = 3.62 \times 10^{-7} m^2 s^{-1} V^{-1}$, $\mu_{Na} = 5.19 \times 10^{-8} m^2 s^{-1} V^{-1}$, $\mu_{OH} = 2.05 \times 10^{-7} m^2 s^{-1} V^{-1}$, $\mu_{Cl} = 7.91 \times 10^{-8} m^2 s^{-1} V^{-1}$. The ion mobility and diffusion coefficient of the $-COO^-$ group are set to zero to represent that this functional group is fixed within the hydrogel network. The -COOH concentrations for different hydrogel compositions are as follows: $C_{R-COOH} = 1.9\, mol/L$ for hydrogel AAc 15 wt%, $C_{R-COOH} = 3.37\, mol/L$ for hydrogel AAc 30 wt%, $C_{R-COOH} = 4.5\, mol/L$ for hydrogel AAc 45 wt%, $C_{R-COOH} = 5.5\, mol/L$ for hydrogel AAc 60 wt%.

In all experiments, we allow ten minutes for the ion concentration inside the hydrogel and the surrounding aqueous environment to reach a steady state before applying the electric actuation signal. To replicate this scenario in the simulation, a static simulation is first conducted to determine the ion concentration distribution without an external electric field. The static simulation results are then used as the initial conditions for the dynamic ion concentration simulation.

**Note 11: Analysis between the 2 μm- and 10 μm-diameter hydrogel cilia**

Our system operates at Re ≈ 0.001 ≪ 1, where inertial effects are negligible. We therefore analyze the dominant actuation force arising from ion migration and the counteracting elastic and viscous forces at different length scales (2 μm vs. 10 μm). Each cilium can be approximated as a pillar of radius $a$ and length $L$, with a fixed aspect ratio $AR = L/a = 18$. To simplify the scaling analysis, we introduce an overall size parameter $s$, representing the characteristic geometric scale of the cilium. Both the radius ($a$) and length ($L$) scale linearly with $s$ (i.e., $a \propto s$, $L \propto s$), while the aspect ratio ($AR = L/a$) remains constant.

**Actuation force (ion migration).**

A closed-form scaling law is difficult to derive directly due to the complexity of the coupled Poisson–Nernst–Planck equations (see main text Methods for details). Instead, we use results from our fully coupled simulations to infer scaling. For step response simulation in DI water:

- $d = 2\ \mu m$ ($L = 18\ \mu m$): tip displacement $y$ ≈ 3.7 μm, actuation force $F_{actuation} \approx 5.65\times10^{-12}$ N.
- $d = 10\ \mu m$ ($L = 90\ \mu m$): tip displacement $y$ ≈ 18.3 μm, actuation force $F_{actuation} \approx 2.1\times10^{-10}$ N.

The force ratio $\frac{2.1\times10^{-10}\ \mathrm{N}}{5.65\times10^{-12}\ \mathrm{N}} = 37$ is close to $s^2$ for a five-fold increase in size, suggesting that the actuation force scales as $F_{actuation} \sim s^2$.

**Viscous resistance**

For small-amplitude bending of a slender filament in Stokes flow, the transverse drag per unit length is

$$\zeta_\perp \approx \frac{4\pi\mu}{\ln(L/a) + 1/2}$$

which is size-independent here because L/a=AR is fixed. For a small angular velocity Ω about the base, the total transverse viscous force scales as

$$F_\perp = \int_0^L \zeta_\perp \Omega x dx = \frac{1}{2}\zeta_\perp \Omega L^2 \quad \Rightarrow F_\perp \sim s^2$$

**Elastic restoring force**

Approximating the ion-driven loading as uniformly distributed along the cilia, the tip-deflection relation for a cantilever gives

$$F_{\text{elastic}} = \frac{8EI}{L^3}y$$

with $I = \pi a^4/4$ and L=AR × a. Hence

$$F_{\text{elastic}} = \frac{2E\pi a}{AR^3}\text{y} \Rightarrow F_{\text{elastic}} \sim sy$$

**Scaling of displacement**

From force balance $F_{actuation} = F_{\perp} + F_{\text{elastic}}$, we obtain

$$\text{y} = \frac{F_{\text{elastic}} \times AR^3}{2E\pi a} = \frac{(F_{actuation} - F_{\perp}) \times AR^3}{2E\pi a} \sim \frac{s^2}{s} \sim s$$

Therefore, tip displacement scales linearly with size, while bending angle (θ ∼ y/L) remains nearly scale-independent. This is confirmed by simulations, where the 2 μm tip displacement ($y \approx$ 3.7 μm) is ~1/5 that of the 10 μm cilium ($y \approx$ 18.3 μm), and by experiments at 5 Hz, where both show similar bending angles (Fig. 2c(iii)).

**Frequency dependence**

At higher frequencies, however, the 10 μm cilium exhibits reduced bending compared to the 2 μm cilium. This can be understood as follows: at low frequency, both cilia reach a "saturated" bending state since ions have sufficient time to redistribute. At higher frequencies, the shorter ion transport path in the 2 μm cilium allows faster response, leading to greater dynamic bending. Step-response simulation sequences (Supplementary Fig. 10) confirm this behavior: within the same time frame, the 2 μm cilium reaches its maximum bending angle earlier, indicating a faster electromechanical response compared to the 10 μm cilium.

## Note 12: Microelectrodes working in high-concentration ionic solutions

In high-concentration ionic solutions, such as physiological saline and DPBS, electrical signals can cause ion accumulation on electrode surfaces. Over time, repeated ion accumulation and diffusion on Pt thin-film electrodes can result in delamination from the polyimide substrate. In physiological saline, these electrodes typically function for tens of minutes. In contrast, in DI

water, the microelectrodes can operate continuously for over 5 hours without noticeable damage. Since fluid experiments usually last several to tens of minutes, a low-concentration NaCl solution (0.00769 mol/L) is used to ensure experimental stability and continuity.

Advanced MEMS fabrication techniques could address the adhesion issues of microelectrodes and enhance their stability in high-concentration ionic solutions. For instance, electrochemical deposition can increase electrode thickness to several micrometers, improving mechanical robustness. High-temperature hot pressing can strengthen adhesion between electrodes and the polyimide substrate, while subsequent annealing relieves internal stresses in the electrode microstructures, further enhancing stability. Alternatively, substrates with stronger adhesion properties may be employed. Future work will explore these advanced microfabrication techniques to improve microelectrode stability.

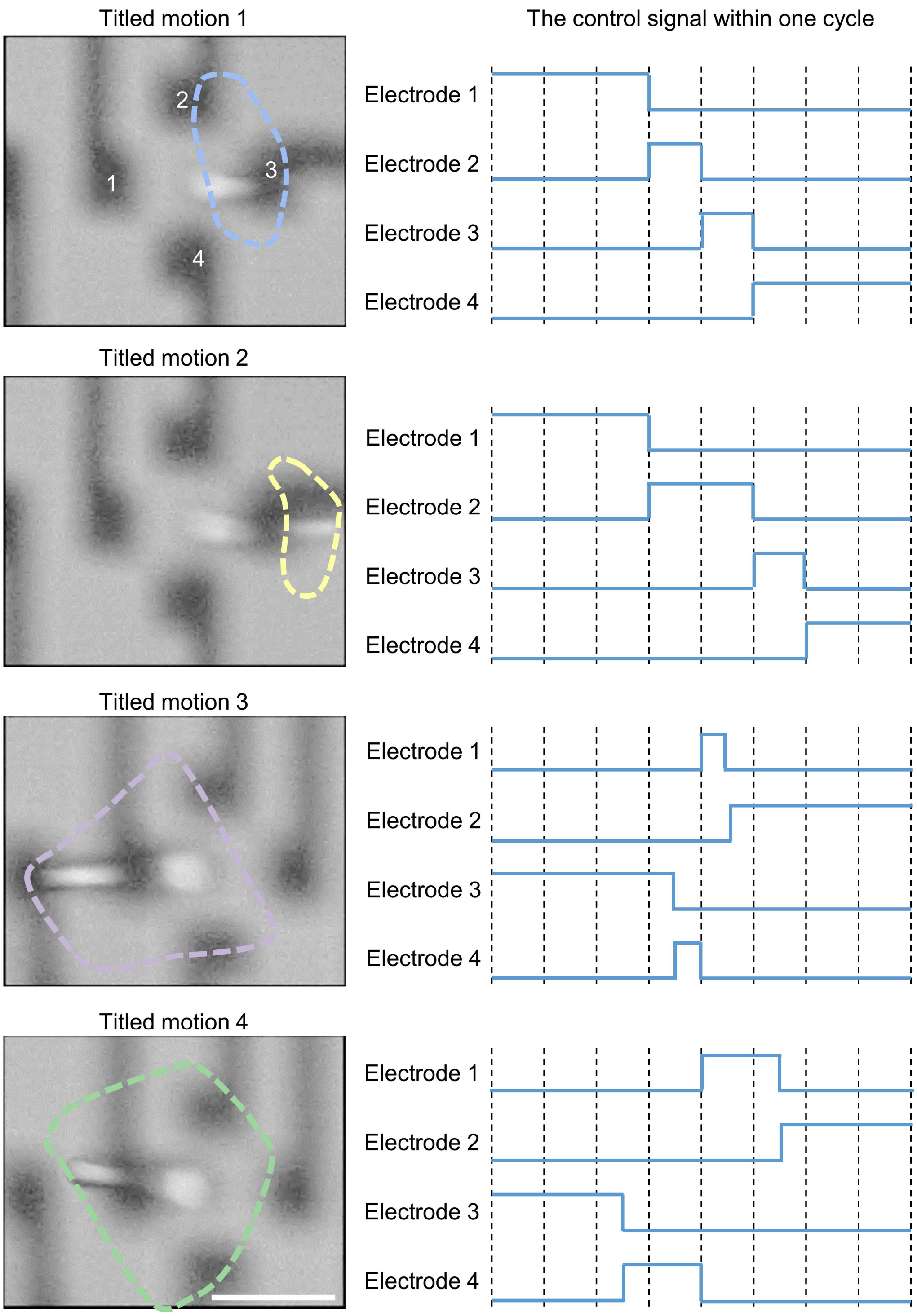


**Supplementary Fig. 1. Reconfigurable motion by adjusting the control signal.** The dashed lines show the motion trajectories and the right column are the corresponding control signals. Scale bar 40 μm.

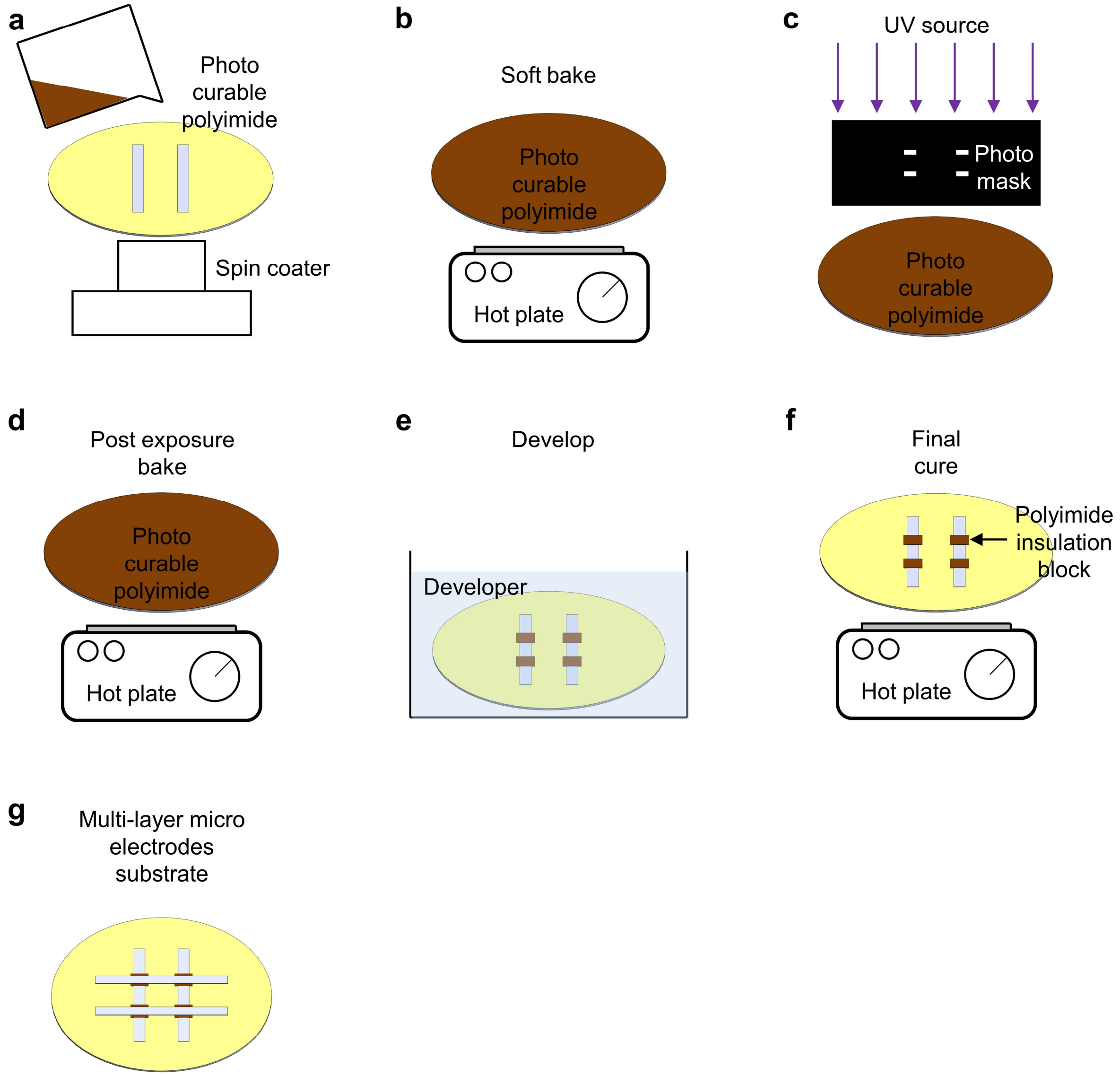


**Supplementary Fig. 2. Multi-layer microelectrodes fabrication process.**

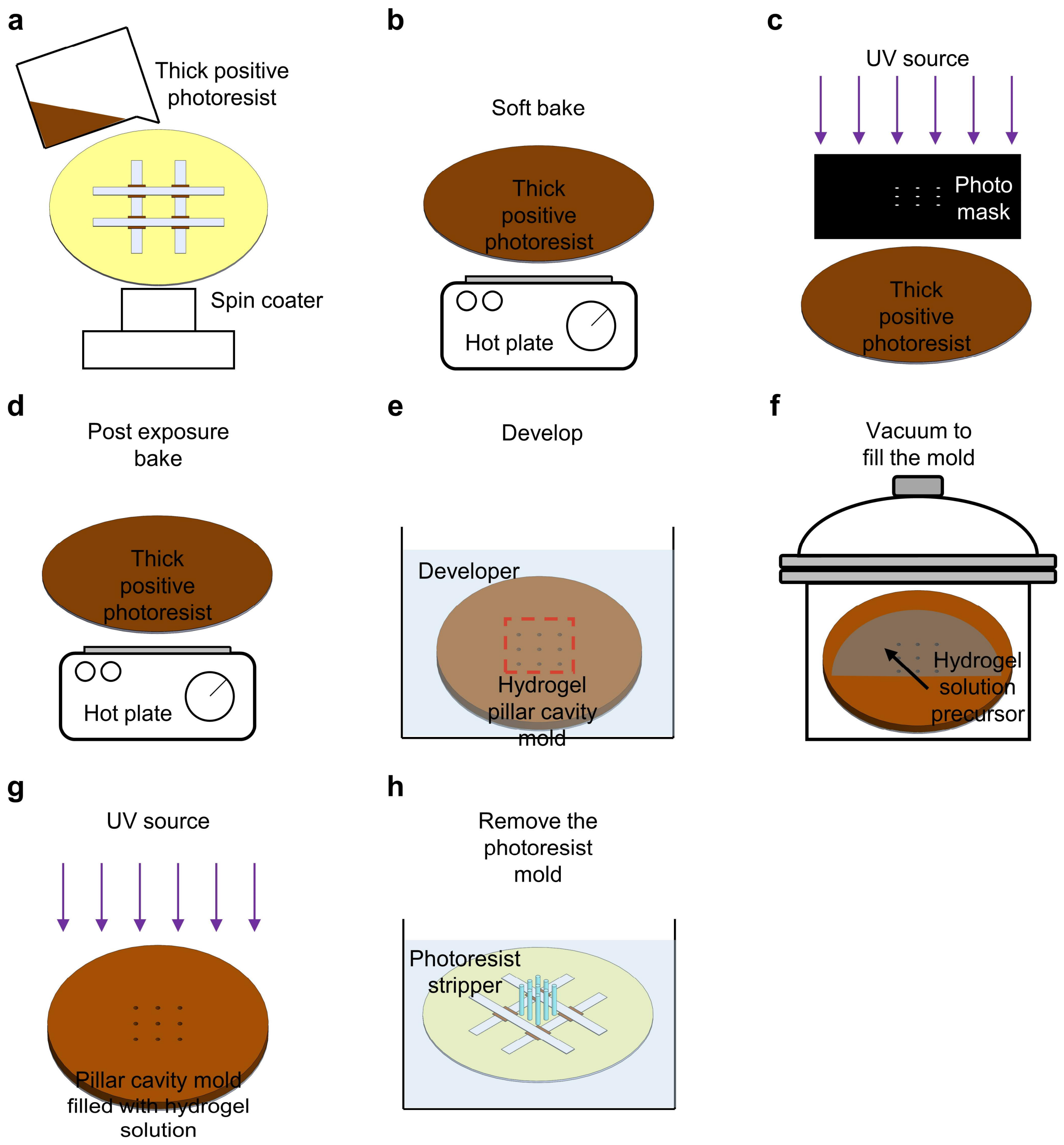


**Supplementary Fig. 3. Gel microcilia fabricated by micro molding.**

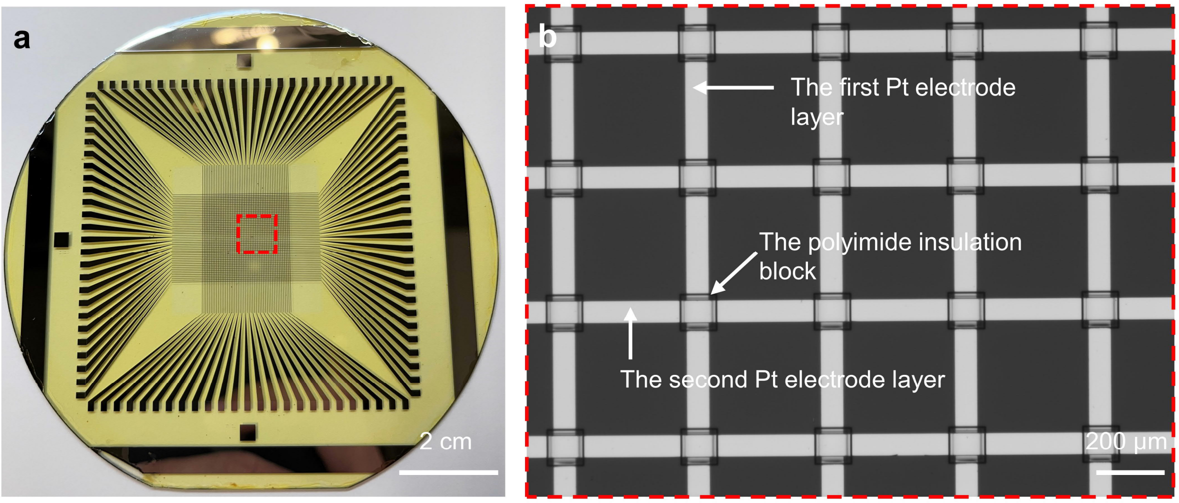


**Supplementary Fig. 4. Multi-layer microelectrodes. a**, The polyimide-based multi-layer microelectrodes on a glass substrate. **b**, Zoomed-in view of the microelectrodes.

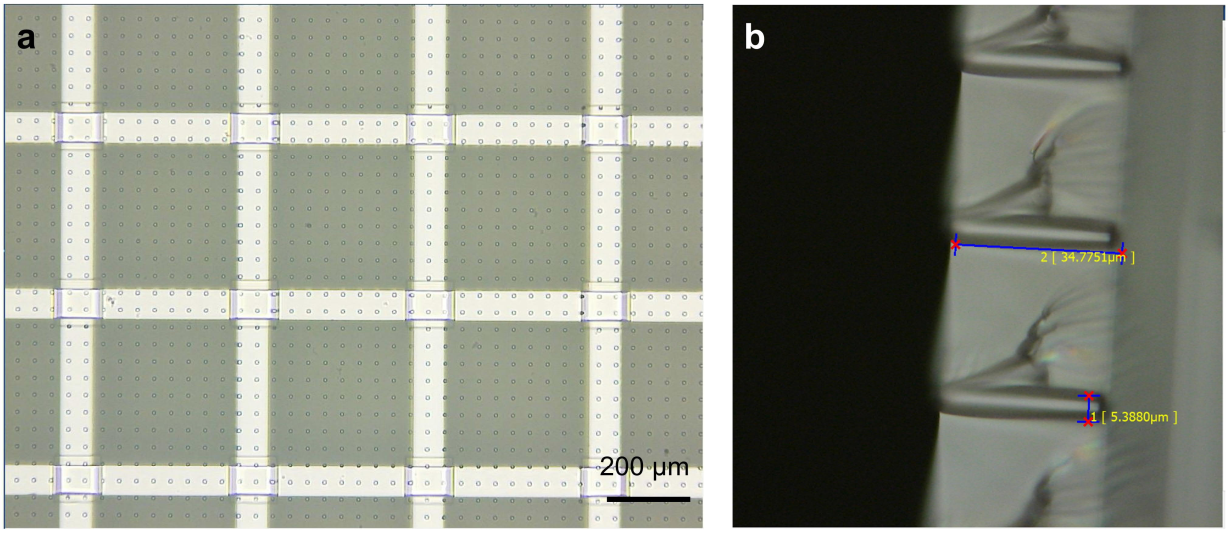


**Supplementary Fig. 5. Multi-layer microelectrodes with gel microcilia photoresist mold. a**, Microelectrodes substrate with dissolvable photoresist mold. **b**, Cross-section view of the photoresist mold.

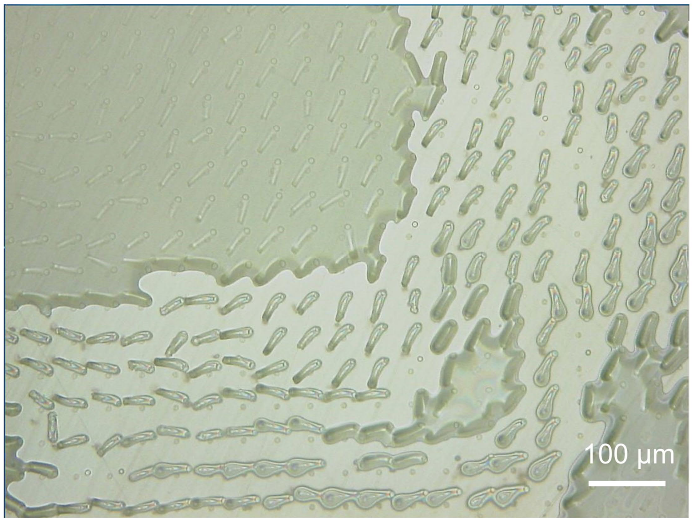


**Supplementary Fig. 6. Retrieved gel microcilia from the photoresist mold.**

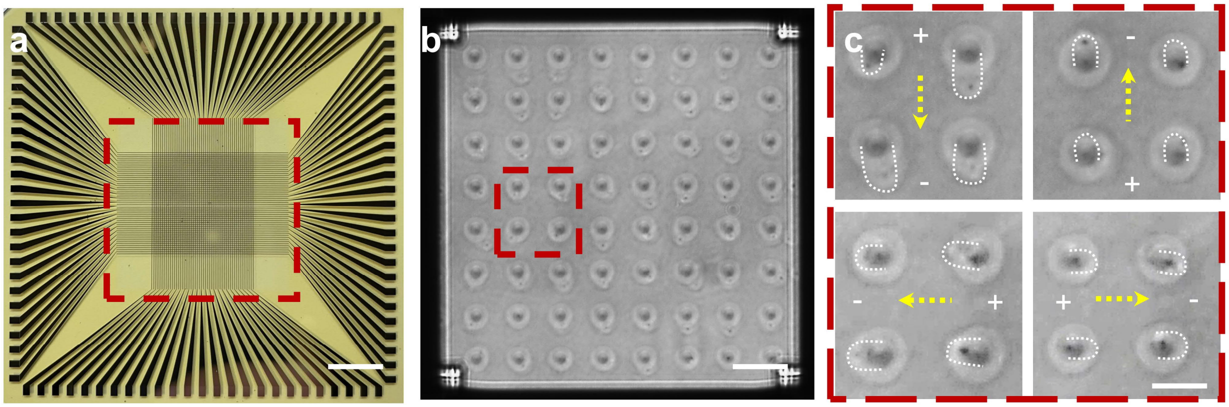


**Supplementary Fig. 7. Reprogrammable motions of a $10^6$ gel microcilia array.** The molded gel microcilia have a diameter of 5 µm and a height of 35 µm. The working solution is DI water. Motion frequency is 2 Hz. **a**, $10^6$ molded gel microcilia array on multi-layer microelectrodes, the microcilia region is marked by the red dashed line. **b**, Zoomed-in view of the molded gel microcilia array within one actuation cell. **c**, Bending motions of four molded gel microcilia. These gel microcilia are from the zoomed-in area highlighted in **b**. The microcilia actuators can be programmed to bend in the *y*-direction or *x*-direction. The microcilia are marked with dashed lines, and arrows indicate the bending directions. Scale bars: **a** 1 cm, **b** 40 µm, **c** 10 µm.

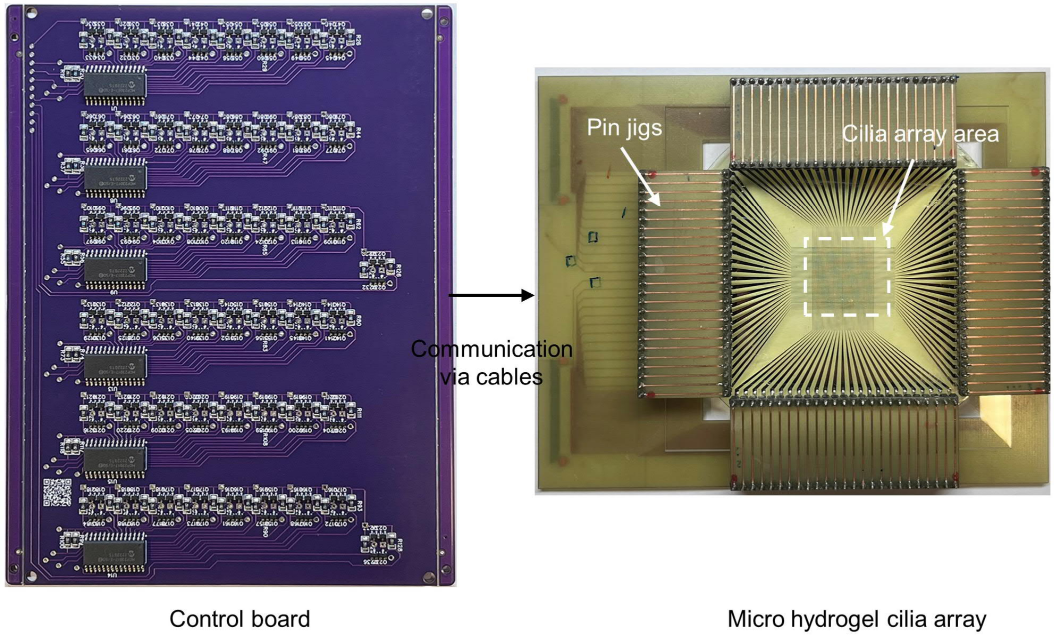


**Supplementary Fig. 8. Control board for gel microcilia actuation.**

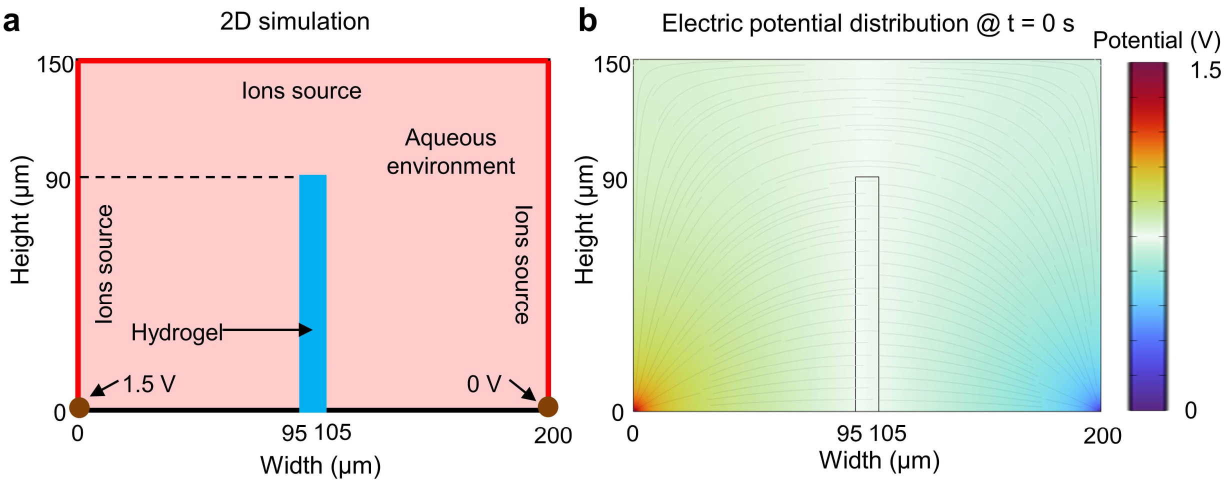


**Supplementary Fig. 9. 2D simulation settings. a**, Ion concentration simulation settings. To reduce simulation time, only the 2D working region (200 µm width × 150 µm height) is simulated instead of the entire bath shown in Extended Data Fig. 6b(i). The hydrogel region is shaded in blue, while the light red area represents the aqueous environment. A voltage of 1.5 V is applied to the 0 µm electrode, and 0 V to the 200 µm electrode. The left, top, and right boundaries (marked by red lines) function as ion sources, simulating the influx of mobile ions from the surrounding solution. **b**, Electric potential distribution at the initial state (t = 0 s).

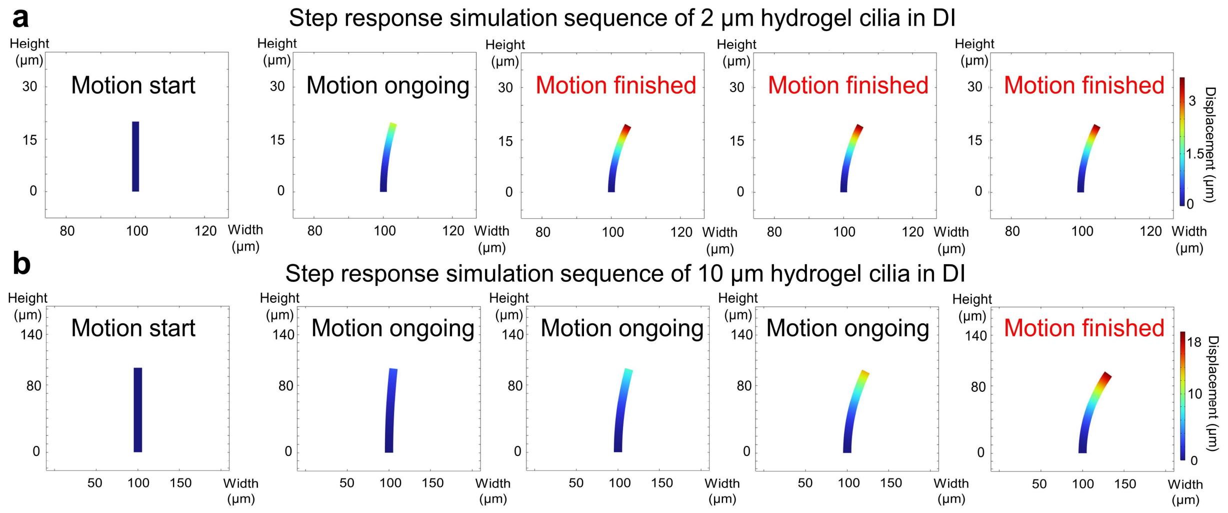


**Supplementary Fig. 10. Simulation comparison between the 2 μm and 10 μm hydrogel cilia.**

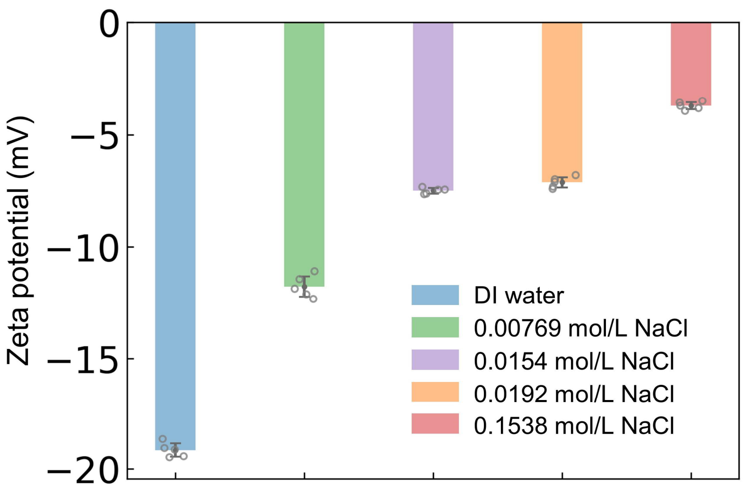


**Supplementary Fig. 11. Zeta potential of 30 wt% AAc hydrogel in different solutions.** In DI water, the negatively charged hydrogel microcilia bent toward the cathode (Fig. 2a). Simulations show that the electrostatic force ($\mathbf{2.5} \times \mathbf{10^{-15}}$ N) is several orders of magnitude smaller than the ion-migration-induced force ($\mathbf{6.16} \times \mathbf{10^{-10}}$ N). Together, the experiments and simulations demonstrate that electrostatic force is negligible in hydrogel microcilia actuation. Data are presented as mean ± s.d. (n = 5 tests).

**Supplementary videos explanations**

**Supplementary Video 1:** Comparison of artificial hydrogel microcilia with biological mouse cilia.

This video sequentially compares the motion of a 10 µm-diameter hydrogel cilia array with that of biological mouse cilia on the ventral surface of the embryonic node, followed by a comparison between 2 µm-diameter hydrogel cilia and their biological counterparts.

**Supplementary Video 2:** Biological cilia reconfiguration.

This video shows the biological cilia's reconfiguration ability.

**Supplementary Video 3**: Hydrogel cilia reconfiguration.

This video shows the artificial hydrogel cilia's reconfiguration ability.

**Supplementary Video 4**: Localized heterogeneous behavior of the cilia.

This video shows the comparison between the biological cilia and the hydrogel cilia. Both show counter-rotating of two adjacent cilia.

**Supplementary Video 5:** Single hydrogel cilium actuation.

This video shows the actuation of a single hydrogel cilium (2 µm diameter, 18 µm height) in physiological saline. It sequentially shows 2D bending, followed by 3D clockwise and counterclockwise rotations (Fig. 1c).

**Supplementary Video 6**: The step response comparison between millimeter hydrogel and micrometer hydrogel.

The video shows the step response difference between millimeter hydrogel and micrometer hydrogel.

**Supplementary Video 7:** The dynamic signal comparison between millimeter hydrogel and micrometer hydrogel.

The video shows difference between millimeter hydrogel and micrometer hydrogel under a dynamic signal.

**Supplementary Video 8**: Hydrogel cilia step response in three typical solutions and the dynamic motion in bio fluids.

The video shows the step response of hydrogel cilia (10 µm diameter, 90 µm height) in three different solutions: DI water, 0.00769 mol/L NaCl, and physiological saline (0.1538 mol/L NaCl) (Fig. 2a-b). Then followed by hydrogel cilia's dynamic motion in human saliva, human serum, and mouse plasma.

**Supplementary Video 9**: The whole process of the hydrogel cilia's step response in DI water.

The whole process of step response in DI water.

**Supplementary Video 10**: The bending simulation video.

The video shows the simulation results of the micro hydrogel bending in DI water, 0.00769 mol/L NaCl, and 0.0154 mol/L NaCl.

**Supplementary Video 11**: Durability test.

The video sequentially shows the bending amplitudes of a hydrogel cilium (10 µm diameter, 90 µm height) after 1,000, 42,000, 78,000, 186,000, and 330,000 continuous actuation cycles (Fig. 2Cii).

**Supplementary Video 12**: Coordinated motions of the two-cilia system.

The video sequentially shows the two hydrogel cilia (2 µm diameter,18 µm height) in physiological saline executing synchronized 2D bending, 2D bending with a 180° phase shift, synchronized 3D rotation, and opposing rotation directions in the two gel microcilia (Fig. 3a).

**Supplementary Video 13**: Motions of the 5 x 5 cilia array.

The video sequentially shows the actuation of a 5×5 hydrogel cilia array (10 µm diameter, 90 µm height) in DI water, including synchronized 2D bending in the *x* and *y* directions, 3D clockwise and counterclockwise rotations, and individually addressable coordinated motions (Fig. 3b(i-x)).

**Supplementary Video 14**: 5 x 5 cilia array - reprogrammable motions to display the "HKUST" letters.

The video shows a 5×5 hydrogel cilia array (10 µm diameter, 90 µm height) in DI water, with reprogrammable motions that form the 'HKUST' letters (Fig. 3b(xi)).

**Supplementary Video 15**: 625 cilia array - reprogrammable motions to display the "MPI-IS" letters.

The video shows a 625 hydrogel cilia array (10 µm diameter, 90 µm height) in DI water, with reprogrammable motions that form the 'MPI-IS' letters (Fig. 3c).

**Supplementary Video 16**: One million cilia array motion.

The video sequentially shows a one million cilia array (5 µm diameter, 35 µm height) in DI water performing synchronized 2D bending in both *x* and *y* directions and a zoomed-in view of the bending motions.

**Supplementary Video 17**: Cilia on 3D surface: right to left flow.

The video shows the cilia could generate a right to left flow on a hill-shaped surface.

**Supplementary Video 18**: Cilia on 3D surface: top to bottom flow.

The video shows the cilia could generate a top to bottom flow on a hill-shaped surface.

**Supplementary Video 19:** Cilia on 3D pyramid frame.

The video shows hydrogel cilia could integrate with a 3D frame structure and perform fluid manipulation on such structure.

**Supplementary Video 20:** Helical-shaped hydrogel actuator.

The video shows the hydrogel could be printed to 3D shapes and demonstrate functions in this 3D configuration.

**Supplementary Video 21:** Biomimetic artificial starfish larva.

The video shows the hydrogel actuator technique could build an artificial starfish larva, and it could generate comparable flows to the biological star larva.

**Supplementary Video 22**. Rotary micromachine array.

The video shows the hydrogel actuator could be integrated with rotary micromachines and actuate them.

**Supplementary Video 23.** Flapping micromachine.

The video shows the hydrogel could be integrated with a flapping micormachine, and the actuator's bending motion could be transferred to a flapping motion via the joint structure.

**Supplementary Video 24**: Fluid Experiment 1.

The video shows four hydrogel cilia (10 µm diameter, 90 µm height) in 0.00769 mol/L NaCl, positioned at the center of each electrode block, generating clockwise vortices that induce a counterclockwise vortex in the inter-block region (Fig. 4a).

**Supplementary Video 25:** Fluid Experiment 2.

The video shows four hydrogel cilia (10 µm diameter, 90 µm height) in 0.00769 mol/L NaCl, positioned at the four corners of each electrode block, generating five vortices per block—four clockwise and one central counterclockwise—with neighboring vortices inducing an inter-block counterclockwise flow (Fig. 4b).

**Supplementary Video 26**: Fluid Experiment 3.

The video shows a dense array of 25 hydrogel cilia (10 µm diameter, 90 µm height) within one electrode block in 0.00769 mol/L NaCl. Hydrodynamic interference cancels intra-block vortices, while peripheral clockwise vortices and a central counterclockwise vortex between blocks emerge (Fig. 4c).

**Supplementary Video 27**: Fluid Experiment 4.

The video shows the fluid manipulation capability of a 5×5 individually controlled cilia array (10 µm diameter, 90 µm height) in 0.00769 mol/L NaCl. Sixteen cilia in the outermost ring rotate clockwise with a 90° phase difference to form metachronal waves, while the remaining cilia remain stationary, producing a centralized counterclockwise vortex (Fig. 4d).

**Supplementary Video 28**: Fluid Experiment 5.

The video shows the fluid manipulation capability of a 5×5 individually controlled cilia array (10 µm diameter, 90 µm height) in 0.00769 mol/L NaCl. A 3×3 cilia array in the top-left corner rotates clockwise, and the remaining cilia rotate counterclockwise, generating clockwise L-shaped flows as confirmed by particle-tracking trajectories (Fig. 4e).

**Supplementary Video 29**: Fluid Experiment 6.

The video shows the fluid manipulation capability of a 5×5 individually controlled cilia array (10 µm diameter, 90 µm height) in 0.00769 mol/L NaCl. Cilia in columns 1, 3, and 5 (15 actuators) rotate clockwise, whereas those in columns 2 and 4 (10 actuators) rotate counterclockwise,

resulting in dominant downward flows between columns 1–2 and 3–4 and upward flows between columns 2–3 and 4–5, as evidenced by particle tracking between columns 3–4 (Fig. 4f).

**Supplementary Video 30**: Fluid Experiment 7.

The video shows the fluid manipulation capability of a 5×5 individually controlled cilia array (10 µm diameter, 90 µm height) in 0.00769 mol/L NaCl. Here, the outermost ring (16 cilia) and the central cilium rotate clockwise, while the middle ring (8 cilia) rotates counterclockwise, establishing nested vortices with counterclockwise flow between the outer and middle rings and clockwise flow between the middle ring and the central cilium, as shown by a circular particle-tracking trajectory between the outer and middle rings (Fig. 4g).

**Supplementary Video 31**: 2D fluid flow simulation.

The video sequentially shows the 2D flow simulations corresponding to fluid experiments 1-7 (Fig. 4).

**References**


1 Odian, G. *Principles of Polymerization*. (2004).

2 Physik Instrumente (PI) SE & Co. KG. P-561 • P-562 • P-563 PIMars Nanopositioning Stage https://www.physikinstrumente.com/en/products/nanopositioning-piezo-flexure-stages/multi-axis-piezo-flexure-stages/p-561-p-562-p-563-pimars-nanopositioning-stage-201550
